# When the Pupils Lie: Unmasking an Unnoticed High Spinal Anesthesia During Orthopedic Surgery

**DOI:** 10.7759/cureus.94047

**Published:** 2025-10-07

**Authors:** Kartik Sonawane, Sumeet Patil, Satheesh Kumar, Tuhin Mistry, Palanichamy Gurumoorthi, Chelliah Sekar

**Affiliations:** 1 Anesthesiology, Ganga Medical Centre and Hospitals, Pvt. Ltd., Coimbatore, IND

**Keywords:** combined spinal-epidural anesthesia, fixed dilated pupils, high spinal anesthesia, intrathecal catheter migration, neurological recovery, patient safety, regional anesthesia complications, sedation monitoring

## Abstract

High spinal anesthesia is a rare but potentially serious complication of neuraxial techniques. Unlike total spinal anesthesia, which typically presents dramatically with profound cardiovascular collapse, high spinal anesthesia may evolve more insidiously and manifest primarily with neurological signs.

We report the case of a 23-year-old polytrauma patient undergoing femur and tibia fixation under combined spinal-epidural anesthesia (CSEA) who developed sudden unresponsiveness with fixed, dilated pupils shortly after femoral canal reaming. An epidural top-up of 10 mL 0.25% bupivacaine was administered 2.5 hours into surgery, with neurological deterioration occurring approximately 30 minutes later. Despite this alarming presentation, his hemodynamics and spontaneous ventilation remained stable, and he regained consciousness within 30 minutes without airway intervention. Postoperative evaluation revealed intrathecal migration of the epidural catheter, with the block extending above T2 and causing bilateral upper limb weakness. Arterial blood gas analysis demonstrated metabolic and respiratory acidosis, reflecting transient hypoventilation.

This case illustrates how an unnoticed high spinal anesthesia can mimic catastrophic neurological or embolic events yet remain fully reversible. It underscores the importance of careful catheter management, structured differential diagnosis, and preparedness for airway intervention.

## Introduction

Combined spinal-epidural anesthesia (CSEA) is commonly used for orthopedic trauma surgeries because it provides the rapid onset and reliability of a spinal block along with the flexibility of prolonged analgesia via an epidural catheter [1,2]. However, epidural catheter migration into the intrathecal space is a recognized, albeit rare, complication that can go unnoticed [3-5]. When local anesthetic (LA) is administered through a misplaced catheter, whether by continuous infusion or incremental top-ups, the spread may extend cephalad, resulting in a high spinal block [3,4].

The risk of epidural catheter migration is well-documented in both cadaveric and clinical studies, with factors such as multiple dural punctures or the size of the Tuohy needle influencing the likelihood of misplacement [4,5]. Additionally, CSEA is associated with a higher incidence of neurological complications compared with spinal or epidural anesthesia alone, although the absolute risk remains low and most deficits resolve spontaneously [1,2,6]. High spinal anesthesia differs from total spinal anesthesia in both onset and severity. High spinal blocks may develop gradually, often presenting with neurological signs such as upper limb weakness, pupillary changes, or transient unconsciousness, sometimes with preserved hemodynamics [3,4]. In contrast, total spinal anesthesia usually results from an accidental bolus of a full epidural dose into the intrathecal space, causing immediate apnea, bradycardia, severe hypotension, and loss of consciousness [4]. While these distinctions are familiar to anesthesiologists, a broader audience, including emergency physicians, trauma surgeons, and perioperative staff, may benefit from a clear comparison of high versus total spinal blocks and their overlapping but distinct clinical features. Accordingly, we have included comparative details throughout this report, along with a differential diagnosis table, to aid clinical reasoning across specialties. 

Intraoperative neurological deterioration in trauma patients complicates the diagnostic process, as other potential causes, such as fat embolism syndrome (FES), air embolism, thromboembolism, or acute cerebrovascular events, must also be considered [7]. Pupillary dilation and unresponsiveness are especially alarming, often prompting concern for catastrophic cerebral injury. This case illustrates how a silent high spinal block can mimic a neurological catastrophe yet resolve spontaneously, underscoring the importance of structured intraoperative reasoning and vigilant clinical monitoring.

## Case presentation

A 23-year-old healthy male sustained multiple injuries following a high-energy collision between a two-wheeler and a four-wheeler. He suffered an open type IIIA fracture of the right femoral shaft, closed midshaft fractures of the tibia and fibula, a medial malleolus fracture, and complex fractures of the right hand, including middle and distal phalanges of the middle finger, a metacarpophalangeal shaft fracture of the ring finger, and combined metacarpophalangeal and proximal phalanx base fractures of the little finger with an associated nailbed injury. Additionally, he had multiple lacerations over the thigh, knee, calf, and heel, which were sutured at the referring hospital. Informed consent for the planned anesthesia technique and its potential complications was obtained prior to surgery.

After obtaining informed consent for the anesthesia plan and potential complications, the patient initially underwent damage control surgery, including wound debridement, primary closure, external fixator application, and K-wire fixation with nailbed repair. Definitive fixation was planned following soft tissue recovery and included removal of the external fixator, retrograde femoral nailing, suprapatellar tibial nailing, and medial malleolus screw fixation.

Preoperatively, the patient received intravenous premedication with midazolam 2 mg and ondansetron 4 mg after securing a wide-bore intravenous cannula. Intraoperatively, all American Society of Anesthesiologists (ASA) standard monitors were applied. CSEA was performed in the sitting position using the needle-through-needle technique with a CSEA set (B. Braun, Melsungen, Germany), consisting of an 18 G Tuohy needle, a 20 G epidural catheter, and a 27 G spinal needle. For the spinal component, 2.8 mL of 0.5% hyperbaric bupivacaine with 25 μg fentanyl was injected intrathecally at the L3-L4 interspace. The 20 G epidural catheter was advanced 4 cm into the epidural space and secured for planned intraoperative supplementation and postoperative analgesia. The procedure was uneventful, resulting in adequate sensory and motor block, ensuring patient comfort and optimal surgical exposure. An epidural catheter was placed for intraoperative supplementation and postoperative analgesia. 

At 2.5 hours into the surgery, an epidural top-up of 10 mL of 0.25% plain bupivacaine was administered after reconfirming negative aspiration using a 10 mL syringe. Simultaneously, a continuous epidural infusion of 0.125% bupivacaine with fentanyl (2 μg/mL) was started at 6 mL/hour via an infusion pump. The patient remained supine throughout, with no head-down or Trendelenburg positioning.

Acute deterioration

During femoral canal reaming, approximately 30 minutes after the epidural top-up, the patient experienced a transient drop in SpO₂ to 92%. Already receiving supplemental oxygen, he was instructed to take deep breaths, and oxygen saturation promptly returned to normal. Shortly thereafter, he became unresponsive to verbal commands and painful stimuli. Pupillary examination revealed bilaterally fixed and dilated pupils, raising immediate concern for catastrophic neurological or embolic events. Despite this alarming presentation, his hemodynamics, including blood pressure (118/76 mmHg) and heart rate (82 beats/min), remained stable, and he continued to breathe spontaneously.

The patient was administered 100% oxygen via tight mask ventilation, and preparations were made for rapid sequence intubation. Simultaneously, bedside point-of-care ultrasonography revealed normal ventricular contractility and no evidence of right heart strain, ruling out massive pulmonary embolism. Capillary blood glucose was normal, and no seizure-like activity was noted. The surgical team was immediately informed of the event and requested to expedite surgical closure, as the procedure was already in its closure stage. Given the preserved spontaneous breathing and stable hemodynamics, the anesthesia team decided to observe closely while remaining fully prepared to secure the airway if deterioration occurred.

Post-event recovery and key diagnostic finding

The total surgical duration was four hours, with an estimated blood loss of 550 mL, and was otherwise uneventful. After approximately 30 minutes, the patient began responding to painful stimuli, opened his eyes to verbal commands, and regained full consciousness. Post-event neurological examination revealed bilateral upper limb weakness and an unexpectedly high sensory block level above T2. Arterial blood gas (ABG) analysis (pH 7.25, PaCO₂ 52 mmHg, PaO₂ 85 mmHg on 100% oxygen, bicarbonate 22 mmol/L, base excess -4, and lactate 2.8 mmol/L) showed combined metabolic and respiratory acidosis, consistent with transient hypoventilation and mild tissue hypoperfusion.

Confirmation of high spinal anesthesia

Suspicion of high spinal anesthesia prompted reassessment of the epidural catheter. While prior aspiration checks using a 10 mL syringe had been negative, re-aspiration with a smaller 2 mL syringe revealed clear cerebrospinal fluid (CSF), confirming intrathecal catheter migration. The catheter was removed, and the patient was closely monitored until full neurological recovery, which occurred within four hours of the onset of symptoms. He was kept under observation and transferred to the ward after 24 hours of stable monitoring, having regained complete motor and sensory functions. Written informed consent was obtained from the patient for publication of this anonymized case report.

The chronological summary of anesthetic events, drug administration, and clinical findings is presented in Table 1.

**Table 1:** Timeline of anesthetic events, drug administration, and clinical findings leading to high spinal anesthesia CSEA: combined spinal-epidural anesthesia, LA: local anesthetic, ABG: arterial blood gas, POCUS: point-of-care ultrasound, SpO₂: peripheral capillary oxygen saturation, CSF: cerebrospinal fluid.

Time since spinal	Event	Dose/intervention	Clinical observation
0 minutes	Spinal administered	2.8 mL of 0.5% hyperbaric bupivacaine + 25 µg fentanyl	T8 sensory level, adequate surgical anesthesia
2.5 hours	Epidural top-up	10 mL of 0.25% plain bupivacaine	No CSF on 10 mL aspiration
Immediately after	Infusion started	0.125% plain bupivacaine + fentanyl (2 µg/mL) @ 6 mL/h	Sensory block maintained
~2.7 hours	Femoral canal reaming	-	SpO₂ dropped to 91%, recovered with deep breaths
~2.8 hours	Sudden unresponsiveness	-	Fixed dilated pupils, bilateral upper limb weakness, hemodynamics stable
+5 minutes	ABG + POCUS	-	Respiratory + metabolic acidosis; normal echo
+20 to 30 minutes	Gradual recovery	-	Pupils reactive, responsive to commands
~3.3 hours	Epidural catheter re-aspirated	2 mL syringe used	Clear CSF confirmed intrathecal migration
Postoperative	-	Catheter removed	Full neurological recovery by 4 hours
24 hours	-	-	Discharged to ward

## Discussion

This case illustrates how an unnoticed high spinal anesthesia can present with dramatic neurological features, including unresponsiveness and fixed dilated pupils, yet spare hemodynamic stability and respiratory drive [3]. The key elements supporting high spinal anesthesia in this case included the use of a CSEA technique with ongoing infusion, unrecognized intrathecal catheter migration, pupillary dilation likely due to brainstem and autonomic involvement, and full neurological recovery as the block regressed [3,8].

In our patient, CSEA was initiated with 2.8 mL of 0.5% hyperbaric bupivacaine and 25 μg fentanyl. At 2.5 hours, an epidural top-up of 10 mL of 0.25% plain bupivacaine was administered, followed by a continuous infusion of 0.125% plain bupivacaine with fentanyl (2 μg/mL) at 6 mL/h. The neurological deterioration occurred approximately 30 minutes after the top-up and correlated temporally with initiation of the infusion and subsequent migration of the epidural catheter into the intrathecal space. The patient remained supine throughout, with no head-down or Trendelenburg positioning, ruling out positional contribution to cephalad spread. The patient’s unexpected neurological decline correlated temporally with the initiation of the infusion and subsequent migration of the epidural catheter into the intrathecal space.

Baricity and total intrathecal dose are critical determinants of cephalad spread. Although hyperbaric bupivacaine (used initially in a controlled dose) tends to settle in dependent areas of the spinal canal, the unintended delivery of 10 mL of plain (isobaric) bupivacaine, followed by a continuous infusion of 0.125% isobaric solution, directly into the intrathecal space likely led to progressive cranial migration of LA. Unlike hyperbaric solutions, isobaric agents can distribute more unpredictably and, in this case, lead to high cervical or possibly brainstem involvement, manifesting as bilateral upper limb weakness, altered sensorium, and fixed dilated pupils.

Intrathecal migration of an epidural catheter is a recognized complication of CSEA [2,3,5,8,9]. In such cases, the catheter inadvertently enters the subarachnoid space, causing delivery of epidural doses of LA directly into the CSF [3,5]. This results in a rapid, high-level block with potential involvement of cervical and even brainstem segments [3]. Risk factors include technical errors during catheter placement, catheter movement during patient positioning or surgical manipulation, and failure to confirm catheter position using aspiration tests or test dosing [3,5,10].

Detection of intrathecal catheter migration is challenging during CSEA, particularly when infusion pumps are running continuously [11]. In our case, catheter placement was initially verified and intermittently rechecked using a 10 mL syringe for aspiration, which did not demonstrate CSF backflow. However, when a high block was suspected based on clinical presentation and ABG changes, re-aspiration using a smaller 2 mL syringe revealed clear CSF, confirming intrathecal migration. The increased negative pressure generated by a smaller syringe enhances the sensitivity of aspiration testing. This highlights the importance of using small-volume syringes (e.g., 2 mL) when verifying catheter position, especially after top-ups or during prolonged infusions, to reliably exclude intrathecal or intravascular misplacement [6,12]. The patient remained supine throughout the case, without any steep head-down tilt, suggesting that the cephalad spread was a function of drug baricity, dose, and migration rather than positioning.

In the present case, the block level extended above T2 and was associated with bilateral upper limb weakness, indicating high cervical spread from a large unintended intrathecal dose of LA [3]. This pathophysiological mechanism explains the profound but reversible neurological findings observed intraoperatively, despite the absence of significant hemodynamic compromise [3,8]. An unusual feature was the absence of hypotension or bradycardia. While high spinal blocks typically produce sympathetic blockade and hypotension, preserved compensatory mechanisms or adequate intravascular volume in young patients may blunt these effects [3,9]. The full neurological recovery over hours and regression of the block confirmed a reversible pharmacological effect rather than a structural brain injury [3,8]. ABG findings of combined metabolic and respiratory acidosis were consistent with transient hypoventilation and mild tissue hypoperfusion during the unresponsive period.

The differential diagnosis for sudden intraoperative unresponsiveness is broad (Table 2). Total spinal anesthesia is characterized by abrupt cardiovascular collapse, apnea, and immediate unconsciousness, typically occurring after a bolus of a full epidural dose into the intrathecal space [9]. This was inconsistent with the preserved hemodynamics and spontaneous ventilation in our patient [3]. FES was considered due to the transient desaturation during femoral reaming; however, it remained unlikely as no petechial rash, tachycardia, or progressive neurological deterioration were observed, and Gurd’s criteria were not fulfilled [7]. Arterial lactate was only mildly elevated (2.8 mmol/L), consistent with transient hypoperfusion rather than systemic embolic compromise. Hypoglycemia and hyperglycemia were ruled out based on normal point-of-care glucose levels, and anaphylaxis was excluded due to the absence of cutaneous signs, bronchospasm, or cardiovascular instability. Air or thromboembolism is typically associated with sudden cardiovascular collapse and right heart strain, which were absent on point-of-care echocardiography [7,9]. Seizure or postictal state was unlikely because there were no convulsions, and neurological recovery paralleled block regression rather than postictal resolution [3,8].

**Table 2:** Differential diagnosis of sudden intraoperative unresponsiveness with fixed pupils during femur nailing ABG: arterial blood gas, ARDS: acute respiratory distress syndrome, ICU: intensive care unit, RV: right ventricle.

Feature	High spinal anesthesia (confirmed)	Total spinal anesthesia	Fat embolism syndrome (FES)	Air/thromboembolism	Seizure/postictal state
Onset	Gradually, after infusion or top-up	Abrupt, immediately after bolus dosing	Delayed, often hours after trauma/reaming	Suddenly, during instrumentation	Sudden
Hemodynamic effects	Often mild or absent; may remain stable	Severe hypotension, bradycardia	Tachycardia, mild hypotension	Profound hypotension, right heart strain	Usually, transient tachycardia or normal
Respiratory status	Hypoventilation, spontaneous breathing preserved	Complete apnea common	Hypoxia, tachypnea, ARDS possible	Hypoxia, circulatory collapse	Usually intact unless a prolonged seizure
Neurological findings	Unresponsiveness, fixed pupils, reversible block	Complete unconsciousness	Confusion, altered sensorium, agitation	Loss of consciousness with poor perfusion	Postictal confusion, drowsiness
Pupils	Dilated, fixed (autonomic blockade)	Dilated, fixed	Usually normal	May be normal or sluggish	Normal
ABG changes	Respiratory + metabolic acidosis (hypoventilation, mild hypoperfusion)	Severe metabolic acidosis	Hypoxemia, respiratory alkalosis	Severe hypoxemia, metabolic acidosis	Usually normal or mild acidosis
Recovery	With block regression (hours)	After airway/ventilation and washout	Slow (days), ICU stay often required	Variable, depends on embolus resolution	Minutes to hours
Key clues	Reversible neurological deficit, high block level	Immediate cardiovascular collapse	Petechiae, long-bone injury timing	Sudden hemodynamic collapse, RV dilatation	Preceded by convulsions

Pupillary dilation in this case was particularly misleading. Fixed, dilated pupils are often interpreted as signs of catastrophic brain injury, but in high spinal anesthesia, transient suppression of parasympathetic activity and unopposed sympathetic tone can produce the same finding [3,7]. Recognizing that such signs may be pharmacologically mediated rather than structural prevented unnecessary escalation, such as emergency neuroimaging or premature airway instrumentation [3,8].

One of the most challenging decisions during this episode was whether to intubate and ventilate the patient or to continue observation. In cases of total spinal anesthesia with apnea or hemodynamic instability, airway control is mandatory. In our patient, however, spontaneous breathing and stable circulation allowed for safe observation while being fully prepared for rapid sequence intubation in case of deterioration [3,7]. This conservative approach was validated by the rapid return of consciousness and complete recovery as the block regressed [3,8].

**Figure 1:**
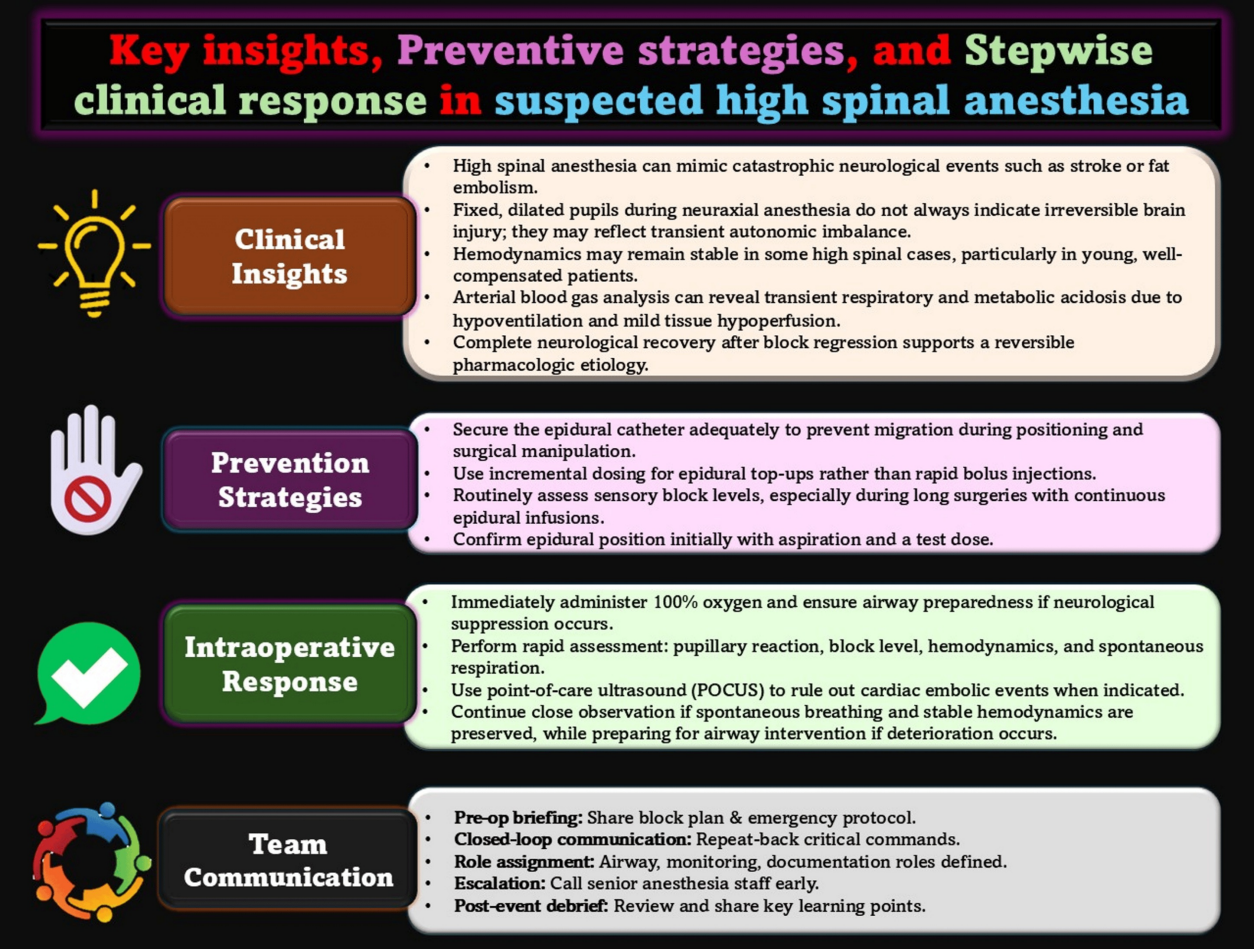
Key insights, preventive strategies, and stepwise clinical response in suspected high spinal anesthesia The figure summarizes contributing factors, warning signs, and recommended actions to guide intraoperative diagnosis and safe management. Image Credit: Kartik Sonawane.

This case emphasizes several learning points (Figure 1): high spinal anesthesia can mimic catastrophic neurological injury yet remain reversible [3,8]; fixed, dilated pupils do not always indicate irreversible brain damage [3,7]; and continuous epidural infusions require frequent sensory level checks to detect excessive cephalad spread early [2,5]. Structured intraoperative reasoning, including point-of-care ultrasonography and ABG analysis, can guide appropriate decision-making and prevent unnecessary interventions [7,8].

Another important consideration highlighted by this case is the role of sedation during orthopedic surgery performed under regional anesthesia (RA). The level of sedation should be carefully titrated to achieve patient comfort while ensuring that the patient remains responsive [2]. A conscious or lightly sedated patient serves as an excellent monitor for the early detection of adverse events, such as unexpected neurological deficits or excessive progression of the neuraxial block [2,9]. In contrast, deep sedation may mask subtle but important clinical signs, such as new-onset limb weakness or altered consciousness, potentially delaying recognition and timely management of critical events [2,9]. Hence, patient responsiveness remains an essential safety tool during RA [2].

Preventing such complications begins with strict vigilance during CSEA. Careful epidural catheter placement with confirmation using aspiration and test dosing can help reduce inadvertent intrathecal placement [2,5]. Incremental dosing, rather than rapid bolus or high-rate continuous infusions, allows for the early detection of unusual block spread [3,5]. Regular assessment of sensory levels is crucial during prolonged surgeries, especially when an epidural pump is in use [2,5]. Additionally, securing the catheter to minimize migration during patient positioning or surgical manipulation is essential [5]. Close intraoperative communication between the anesthesia and surgical teams can facilitate early detection of unusual neurological or physiological changes [2,9].

The strengths of this report include a detailed description of intraoperative reasoning, the integration of point-of-care echocardiography to rule out cardiac embolic events, and a structured follow-up that confirms complete neurological recovery. The inclusion of arterial blood gas findings and sensory level assessment adds objective support to the final diagnosis of high spinal anesthesia. The clinical scenario also provides a teaching point that fixed, dilated pupils do not always signify irreversible brain injury, which may help avoid unnecessary invasive interventions or premature airway instrumentation in similar situations.

This case report is limited by the absence of confirmatory imaging (CT or MRI brain) to rule out embolic or structural brain injury completely. However, the rapid neurological recovery and correlation with regression of block strongly support a neuraxial cause rather than a primary central nervous system event. Moreover, the diagnosis of inadvertent intrathecal catheter migration was confidently established by aspiration of clear CSF using a 2 mL syringe, a technique known to generate higher negative pressure and improve sensitivity for detecting intrathecal placement. Since the catheter was immediately removed upon confirmation, and the patient exhibited no persistent neurological deficits, post-event imaging, such as CT, myelography, or MRI, was deemed unnecessary. This conservative but structured approach helped avoid unnecessary investigations in a clinically stable patient. Another limitation is the lack of intrathecal pressure monitoring, which could have provided objective evidence of catheter migration at the time of event recognition.

## Conclusions

Unnoticed intrathecal migration of an epidural catheter during CSEA can result in high spinal anesthesia, producing dramatic neurological manifestations while sparing cardiovascular function. This case highlights the importance of vigilance, regular block assessment, and structured differential diagnosis when unexpected intraoperative unresponsiveness occurs. Pupillary dilation in such scenarios does not always indicate catastrophic neurological injury and may simply represent a transient pharmacological block. Preventive measures, including careful catheter placement and frequent intraoperative monitoring, remain essential for safe anesthetic practice.
